# Fractionating of Calcium in Tuber and Leaf Tissues Explains the Calcium Deficiency Symptoms in Potato Plant Overexpressing *CAX1*

**DOI:** 10.3389/fpls.2019.01793

**Published:** 2020-01-31

**Authors:** Hongbo Gao, Xiaolei Wu, Cinthya Zorrilla, Sandra E. Vega, Jiwan P. Palta

**Affiliations:** ^1^ College of Horticulture, Hebei Agricultural University, Baoding, China; ^2^ Department of Horticulture, University of Wisconsin-Madison, Madison, WI, United States

**Keywords:** potato, *CAX1*, calcium contents, calcium oxalate, water soluble calcium, acid soluble calcium, *Solanum tuberosum*

## Abstract

Consistent with reports on other plants we recently reported that a potato transgenic line (AT010901) overexpressing *sCAX1* show classic symptoms of calcium deficiency shoot tip injury, leaf curling, leaf margin necrosis and tuber internal defects such as hollow heart and brown spots. The present study was undertaken to quantify calcium in various fraction of leaf and tuber tissues of this transgenic and wild type potato clones to understand the development of these deficiency symptoms at normal calcium nutrition (1mM) and its mitigation at higher calcium nutrition (10mM). Plants were grown in controlled environment growth chamber and watered with balanced nutrient solution containing either 1 or 10 mM calcium. The plants overexpressing *sCAX1* showed calcium deficiency symptoms while sequestering calcium in the vacuole as calcium oxalate crystals. Various fractions of calcium were qualified in the young and mature leaves as well as tuber tissue. A reduced concentration of water soluble fraction of calcium was most important factor related to the development of calcium deficiency symptoms in the line overexpressing *sCAX1*. Furthermore, an increase in this fraction appear to explain the alleviation of the deficiency symptoms in these transgenic plants.Ours is the first study to document the significance of water-soluble calcium in the development of calcium-deficiency symptoms in the potato transgenic lines overexpressing *sCAX1*. Furthermore, our result demonstrates that an increase in this fraction plays a significant role in the alleviation of calcium deficiency symptoms when calcium concentration in the nutrient media is increased. These results provide important insight on the role of *sCAX1* in the calcium homeostasis.

## Introduction

As an essential plant macro-nutrient, calcium plays a vital role in plant growth and human health. Plants use Ca^2+^ to strengthen cell walls, neutralize vacuolar anions, and provide protection against stress (Palta, 1996; Busse et al., 2008; Tang and Luan, 2017). If the level of calcium associated with membranes is reduced, those membranes can become leaky, resulting in the loss of cellular salt and organic compounds (Arora and Palta, 1988; Palta, 2010). By binding pectins in the middle lamella, calcium is also a component of the cell wall and is essential for strengthening its structure (Matoh and Kobayashi, 1998; Cosgrove, 2005). Calcium antiporters and efflux pumps are important for maintaining cytoplasmic calcium at low levels and for restoring normal Ca^2+^ levels after perturbation (Tuteja and Mahajan, 2007; Shao et al., 2008). However, calcium moves with water in the xylem, and very little water moves to tuber and fruit tissues compared to leaf tissue. As a result, calcium concentrations are much greater in foliage than in tubers or fruit, which can cause blossom-end rot or tuber internal defects (Kleinhenz et al., 1999; Chung et al., 2010).

Biofortification represents a powerful tool for greatly enhancing the nutritional value of food crops worldwide. The H^+^/Ca^2+^ antiporter *CAX1* is a tonoplast calcium antiporter that has been identified and cloned from *Arabidopsis thaliana via* yeast suppression mutants defective in vacuolar Ca^2+^ transport (Hirschi et al., 1996). When *CAX1* is expressed in yeast, the N-terminal regulatory region of *CAX1* protein acts as an autoinhibitory domain of H^+^/Ca^2+^ transport activity (Pittman et al., 2002). This region was removed to generate a deregulated short version, which was termed cation exchanger 1 (*sCAX1*) (Cheng et al., 2005). Owing to their ability to store calcium in plant vacuoles, multiple Ca^2+^/H^+^ antiporters have been described and proposed to increase the calcium content in various crop species. *CAX1* has been recognized as a key regulator of apoplastic Ca^2+^ concentrations, and its product maintains low apoplastic Ca^2+^ levels *via* compartmentation into mesophyll vacuoles, which is essential for sufficient plant function and productivity (Conn et al., 2011). The vacuole is known to serve as the reservoir for excess accumulation of Ca^2+^ (Robertson, 2013).The over-expression of *sCAX1* in carrot (Park et al., 2004), tomato (Park et al., 2005a) and potato (Park et al., 2005b) has been shown to increase calcium contents in the edible parts of those species. Some studies showed that this over-expression can result in calcium deficiency symptoms such as blossom-end rot in tomato fruit (de Freitas et al., 2011) and leaf necrosis in tobacco (Hirschi, 1999).

Recently our laboratory has reported similar results for transgenic potato plants over-expressing s*CAX1* (Zorrilla et al., 2019). In this study one of the transgenic line (AT010901) was shown to have calcium deficiency symptoms in leaf and tuber tissues at normal calcium concentration in the nutrient media while the wild type did not show ant deficiency symptoms. The transgenic line was found to sequester calcium in the form of calcium oxalate in the vacuole. Furthermore, these deficiencies in AT010901 were mitigated when plants were grown with much higher calcium concentration in the nutrient solution (Zorrilla et al., 2019). The present study was undertaken to quantify calcium in various fraction of leaf and tuber tissues of this transgenic and wild type potato clones to understand the development of these deficiency symptoms at normal calcium nutrition and its mitigation at higher calcium nutrition. By qualifying different fractions of calcium in the transgenic line overexpressing *sCAX1* we demonstrate that water soluble fraction plays most important role in the development of calcium deficiency symptoms in these transgenic plant at normal root zone calcium as well as in the alleviation of these symptoms at the high root zone calcium.

## Materials and Methods

Experiments were conducted at the University of Wisconsin-Madison using a transgenic line of potato cultivar `Atlantic' designated as AT010901 and the corresponding wild type. This transgenic line carried the cdc2a promoter cdc2a::*sCAX1*and a single copy of *sCAX1* (Zorrilla et al., 2019). The cdc2a::s*CAX1* construct was kindly provided by Dr Kendall Hirschi. The plants were grown *in vitro* from single-node stem sections on Murashige and Skoog (MS) media under continuous light at 20 ± 2°C for 4~5 weeks. Twelve micropropagated seedlings with 5-7 nodes from each genotype were transplanted into 2.5 L plastic pots that contain Metro-Mix (Canada) media in a growth chamber. The plants were cultured under air temperatures of 20°C/15°C (day/night), ambient relative humidity, and 330~350 µmol m^-2^ s^-1^ photosynthetic photon flux density supplied by cool-white fluorescent lamps (16 h photoperiod). The plants were initially given distilled H_2_O during the first 5 days then fertilized once daily with 300 ml of 1/2-strength modified Hoagland solution, which was made with distilled H_2_O. After one month, the plants of each genotype were divided into two parts that received either 10 mM or 1 mM calcium. The calcium was added in the form of CaCl_2_ to the balance the Hoagland solution. Each treatment comprised six independent replicates using a randomized block design with different calcium treatments. When the plants were sampled at 3 weeks after the treatments were applied, the aboveground parts and tubers of 6 plants per treatment were collected and weighed. We collected leaves and tubers (five replications) to determine the different calcium concentrations, and we also observed the calcium oxalate accumulation in the leaves of different varieties subjected to 1 and 10 mM calcium treatments.

### Calcium Extraction and Quantification

Both young (second and third expanded leaves) and mature leaves (fifth fully expanded leaves) were sampled and freeze dried immediately. At the same time, tubers were sampled and freeze dried. These freeze dried tissues were used for the quantification of water-extractable calcium, HCl-extractable calcium and total calcium.

The total calcium in the tissue was extracted and quantified according the method of Kratzke and Palta (1986). The new leaf and old leaf powder samples were ashed at 550°C. The ashes were dissolved in 5 ml of 2 N HCl and filtered using acid-treated filter paper. The solutions were collected in a 50 ml volumetric flask that contained 10 ml of LaCl_3_ (2,000 mg L^-1^ solution) in advance. The final volume was set to 50 ml. The calcium concentration was determined using a Varian SpectrAA 55B atomic absorption spectrophotometer (Varian Instruments inc., Walnut Creek, CA, USA) and was expressed in micrograms per gram of dry weight (DW).

With respect to the water-extractable calcium, approximately 0.1 g of dry leaf powder and 1.0 g of tuber powder was mixed with 20 ml of distilled-deionized water, stirred thoroughly and incubated overnight at 4°C. To recover water soluble calcium, the mixture was filtered using an acid treated filter paper and 12.5 ml of the filtrate was mixed with 12.5 ml of H_2_O_2_ to oxide organic compounds in the filtrate. To insure complete oxidation of organic compounds the mixture was incubated at room temperature for a week. To this mixture 5 ml of 2N HCl and 10 ml of 2,000 mg L^-1^ LaCl_3_ was added with a final volume of 50ml using distilled water. Same procedure was use for extraction of HCl soluble calcium using 0.1 N HCl instead of water in the first step of extraction. Calcium concentration was quantified using Varion Spectra AA 55B as above for the total calcium.

For determination of apoplastic calcium approximately 2.0 g of mature leaf tissue (fourth and fifth fully expanded leaves) was collected from the plants. After removing the midrib, 2-3 mm thick slices of the leaf were made perpendicular to the midrib. The slices were rinsed quickly with distilled-deionized water to remove sap from the cut ends and subsequently placed in a test tube that contained 20 ml of 0.5 mol L^-1^ mannitol. The tube was then placed under vacuum for 10 minutes to facilitate the penetration of mannitol solution in the leaf tissue and shaken for three h at 220 rpm. After shaking 15 ml of the solution was mixed with 15 ml of hydrogen peroxide and incubated at room temperature for 7 days to oxidise the organic compounds. After 7 days, 5 ml of HCl and 10 ml of LaCl_3_ were added to this mixture and finally distilled water was added to make the final volume of 50 ml. The calcium concentration was determined by atomic absorption spectroscopy using a SpectrAA 55B instrument as described above.

### Observations of Calcium Oxalate in Potato Leaves

New leaves (second and third fully expanded leaves) and mature leaves (fifth fully expanded leaves) were sampled separately to observe calcium oxalate crystals under a microscope. A thin layer of vascular tissue was peeled off from the abaxial surface of the midrib using small forceps and then placed immediately in a drop of distilled-deionized water. The sample was submerged in distilled-deionized water at room temperature and subjected to vacuum for 5 minutes to remove air from the sample. Calcium oxalate crystals were observed using an Olympus epifluorescence microscope equipped with polarizer.

### Statistical Analysis

The effects of various treatments were analysed by the SAS 8.1 statistical program (SAS Institute, Cary, NC) using Fisher's least significant difference (LSD) in conjunction with one-way ANOVA at a 0.05 level of significance.

## Results

### Effects of Calcium on the Growth of Wild-Type “Atlantic” and Transgenic Plants

**Figure 1:**
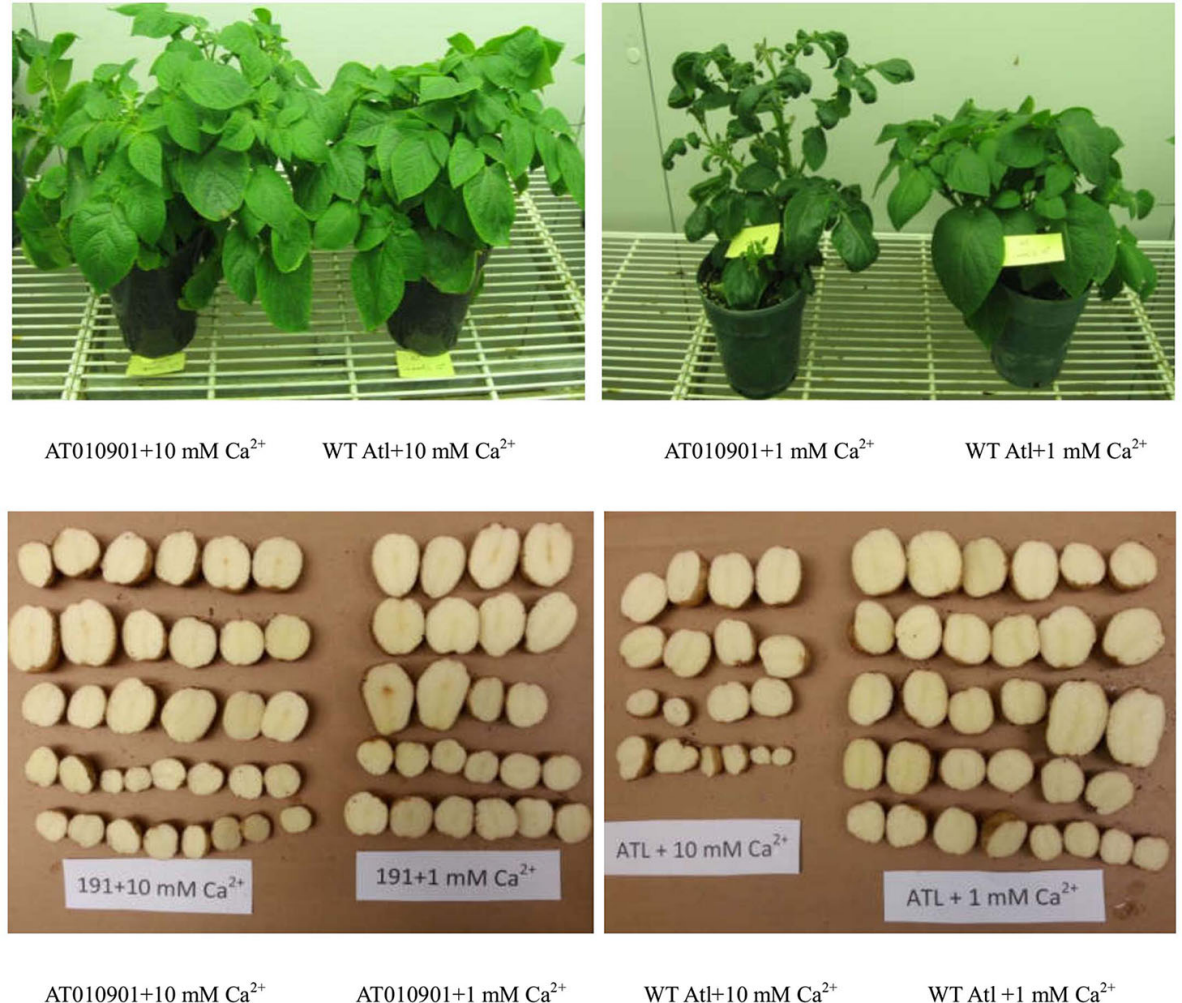
The growth and tubers of wild-type “Atlantic” (WT Atl) and “AT010901” transgenic plants under high-calcium (10 mM) and low-calcium (1 mM) treatments.

**Figure 2:**
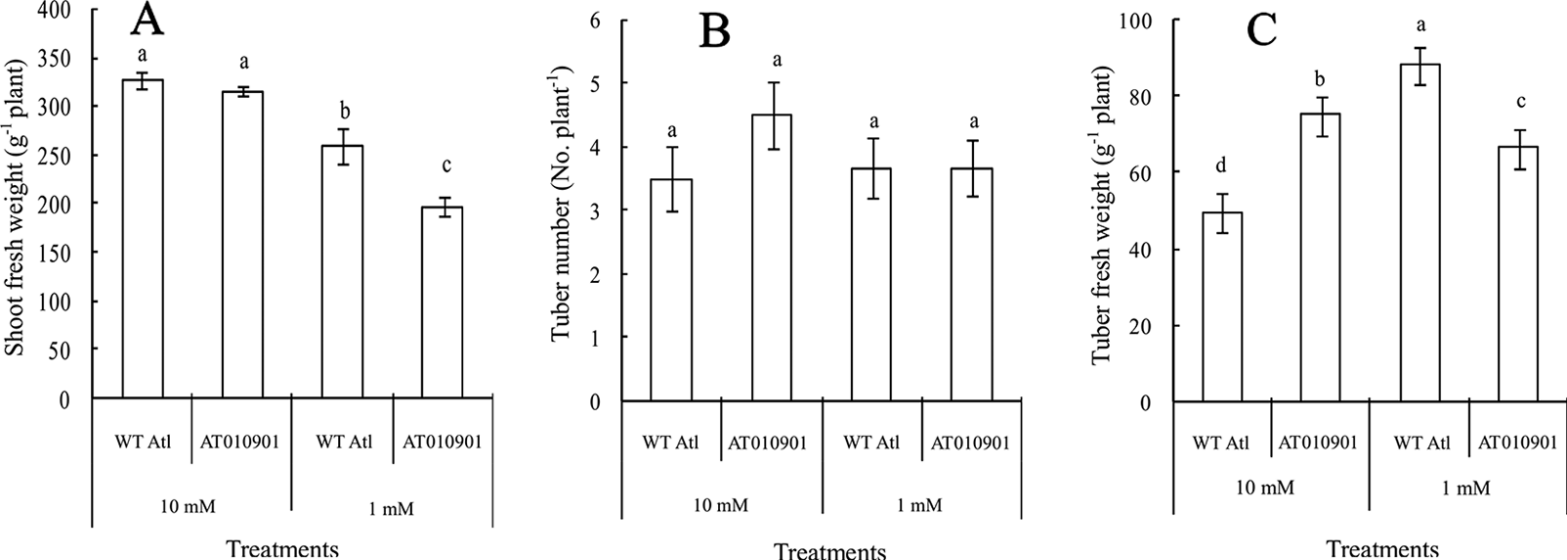
Comparison of shoot growth, tuber weight, and tuber number between wild-type “Atlantic” plants (WT Atl) and “AT010901” transgenic plants under high-calcium (10 mM) and low-calcium (1 mM) treatments as well as the effects of high calcium (10 mM) and low calcium (1 mM) on the shoot fresh weight **(A)**, tuber number **(B)**, and tuber fresh weight **(C)** of both types of plants for 3 weeks. The diameter of each tuber was larger than 1 cm. Each histogram data point represents the mean value of six individual plants, and the vertical bars indicate the SEs (n = 6). The different letters indicate statistically significant differences among the mean values of both types of plants (P-value < 0.05).

There was a significant difference in shoot growth and tuber morphology between wild-type and transgenic plants treated with 10 mM and 1 mM Ca for 3 weeks (**Figures 1** and **2**). The ‘AT010901’ transgenic plants exhibited severe calcium deficiency symptoms under 1mM treatment, such as apical shoot damage and leaf margin necrosis, especially in the young leaves, as well as symptoms of tuber hollow heart. However, these symptoms of calcium deficiency were not observed for plants grown under the 10 mM calcium treatment. The wild-type “Atlantic” plants displayed no symptoms of calcium deficiency under the 1 mM or 10 mM calcium treatment (**Figure 1**).

After three weeks of calcium treatments there was a significant reduction in fresh weight of transgenic plant as compared to the wild-type under 1 mM calcium treatment (**Figure 2**). There were no significant differences in the shoot fresh weight between the two types of plants under the 10 mM calcium treatment (**Figure 2A**). Moreover, no significant difference in tuber number was observed among the four treatments (**Figure 2B**). However, the tuber weight of the transgenic plants was higher than that of the wild-type plants under the 10 mM conditions but was lower under 1mM treatment (**Figure 2C**).

### Changes in Calcium Contents in the Tissue of the Young Leaves of Wild-Type “Atlantic” and Transgenic Plants

**Figure 3:**
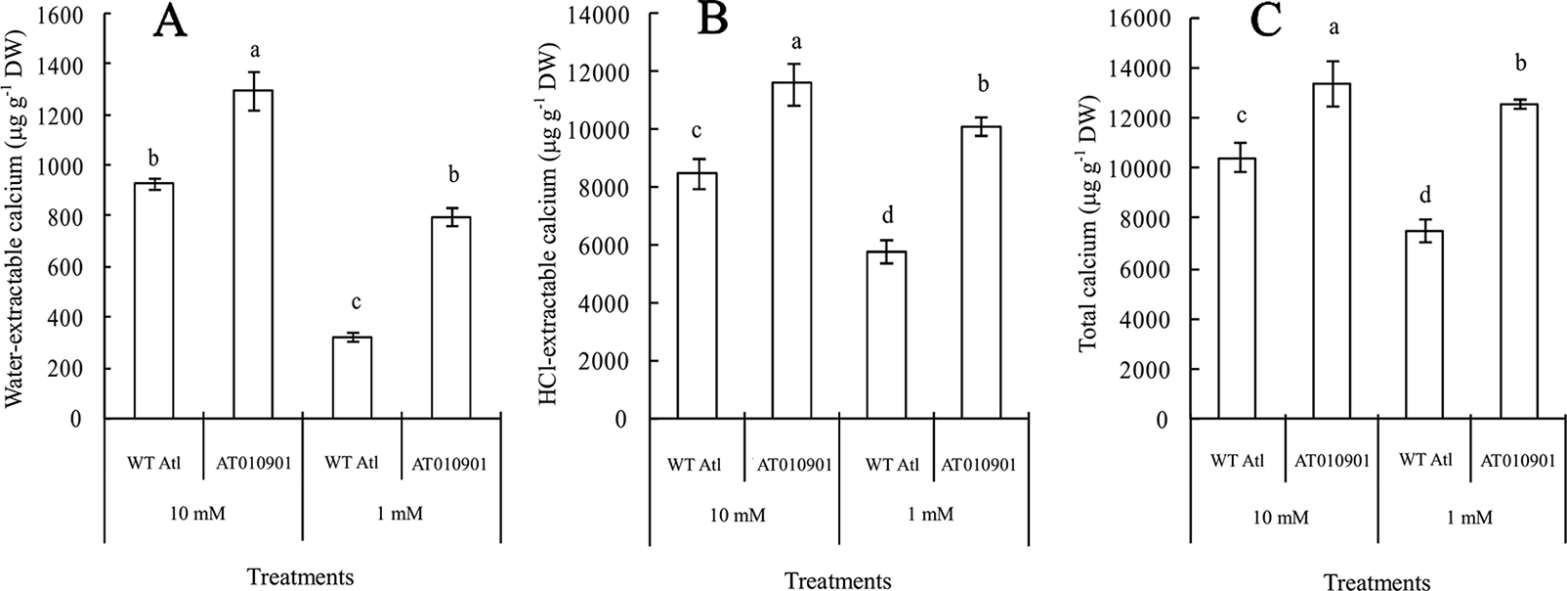
Effects of exogenous calcium on the contents of water-extractable calcium **(A)**, HCl-extractable calcium **(B)** and total calcium **(C)** in the young leaves (second and third expanded leaves) of wild-type “Atlantic” plants (WT Atl) and “AT010901” transgenic plants under high-calcium (10 mM) and low-calcium (1 mM) treatments for 3 weeks. The individual data points represent the means ± SEs (n = 5) of five different plants.

**Figure 4:**
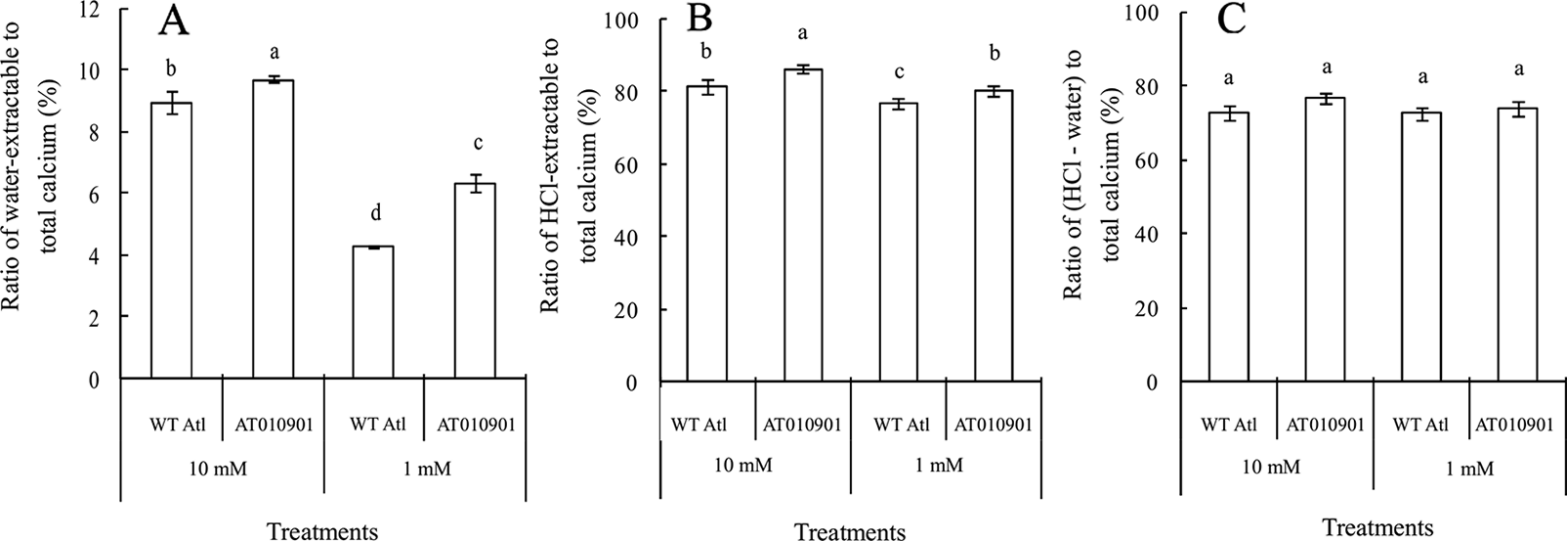
Effects of exogenous calcium on water-extractable calcium/total calcium **(A)**, HCl-extractable calcium/total calcium **(B)** and (HCl-extractable calcium - water extractable calcium)/total calcium **(C)** ratios of the young leaves (second and third expanded leaves) of wild-type “Atlantic” plants (WT Atl) and “AT010901” transgenic plants under high-calcium (10 mM) and low-calcium (1 mM) treatments for 3 weeks. The individual data points represent the means ± SEs (n = 5) of five different plants.

With respect to the young leaves (the second and third expanded leaves from the top), the contents of different types of calcium were measured after 3 weeks under 10 mM or 1 mM calcium treatment (**Figure 3**). The results showed that as compared to wild type the ‘AT010901' transgenic plants had the higher level of water-extractable calcium, HCl-extractable calcium and total calcium under both 1mM and 10 mM calcium treatments (**Figure 3**).The water soluble fraction of the calcium was very small whereas the HCl soluble fraction was nearly 80% of the total calcium in the young leaves (**Figure 4**). All fractions of calcium increased when nutrient treatment was increased from 1 to 10 mM calcium. However, the water soluble fraction increased the most, 62.5% as compared with the HCl and total fraction which increased 14.6% and 6% respectably when treatment solution was increased from 1 to 10 mM (**Figure 4**).

### Changes in Calcium Contents in the Tissue of Mature Leaves of Wild-Type “Atlantic” and Transgenic Plants

**Figure 5:**
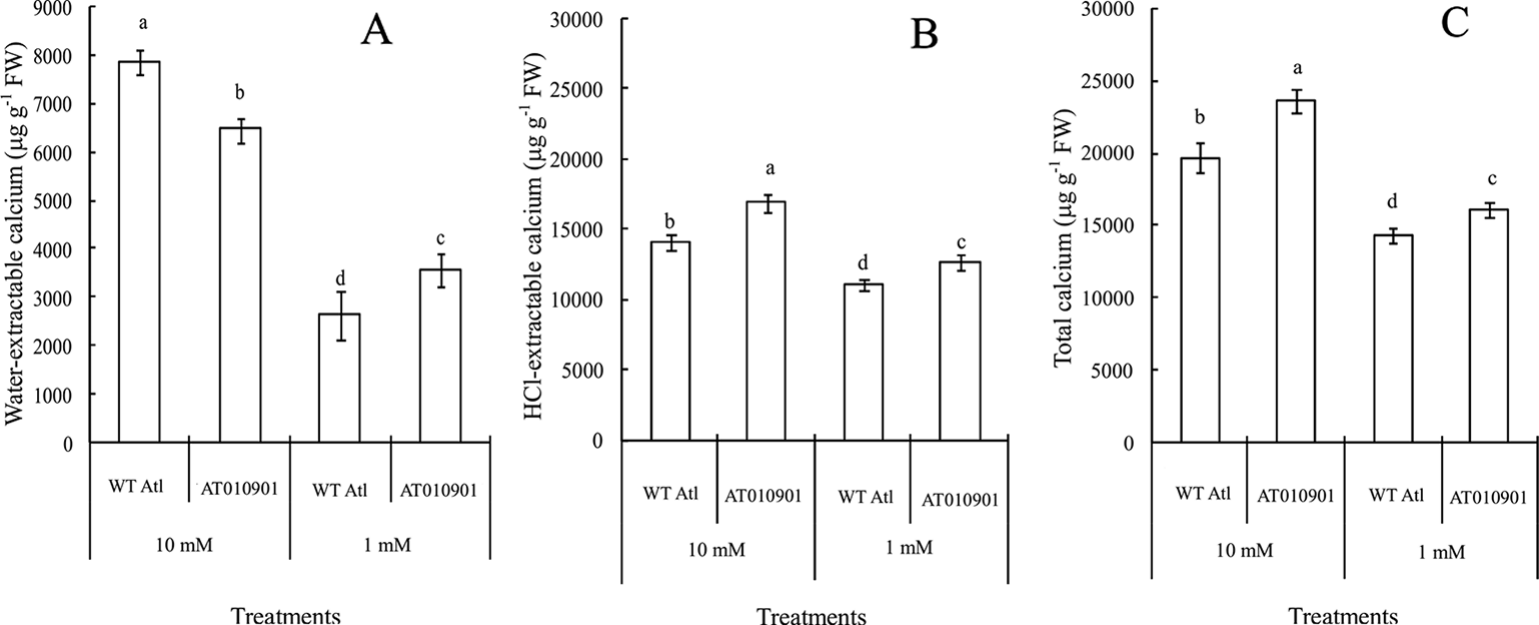
Effects of exogenous calcium on the contents of water-extractable calcium **(A)**, HCl-extractable calcium **(B)** and total calcium **(C)** in the mature leaves (fifth fully expanded leaves) of wild-type “Atlantic” plants (WT Atl) and “AT010901” transgenic plants under high-calcium (10 mM) and low-calcium (1 mM) treatments for 3 weeks. The individual data points represent the means ± SEs (n = 5) of five different plants.

With respect to mature leaves (the fifth fully expanded leaves), the changes in calcium contents differed from those in the young leaves of wild-type and transgenic plants after 3 weeks of 10 mM and 1 mM calcium treatment (**Figure 5**). Compared with the 1 mM calcium treatment, the calcium sufficiency (10 mM) treatment significantly enhanced the water-extractable calcium, HCl-extractable calcium and total calcium in the mature leaves of both wild-type and transgenic plants. This increase was more dramatic in water soluble fractions (**Figure 5A**) than in the other fractions. For example water soluble fraction increased by 81.7% as compared to HCl soluble and total calcium which increased by 33.7% and 46.4% respectably.

**Figure 6:**
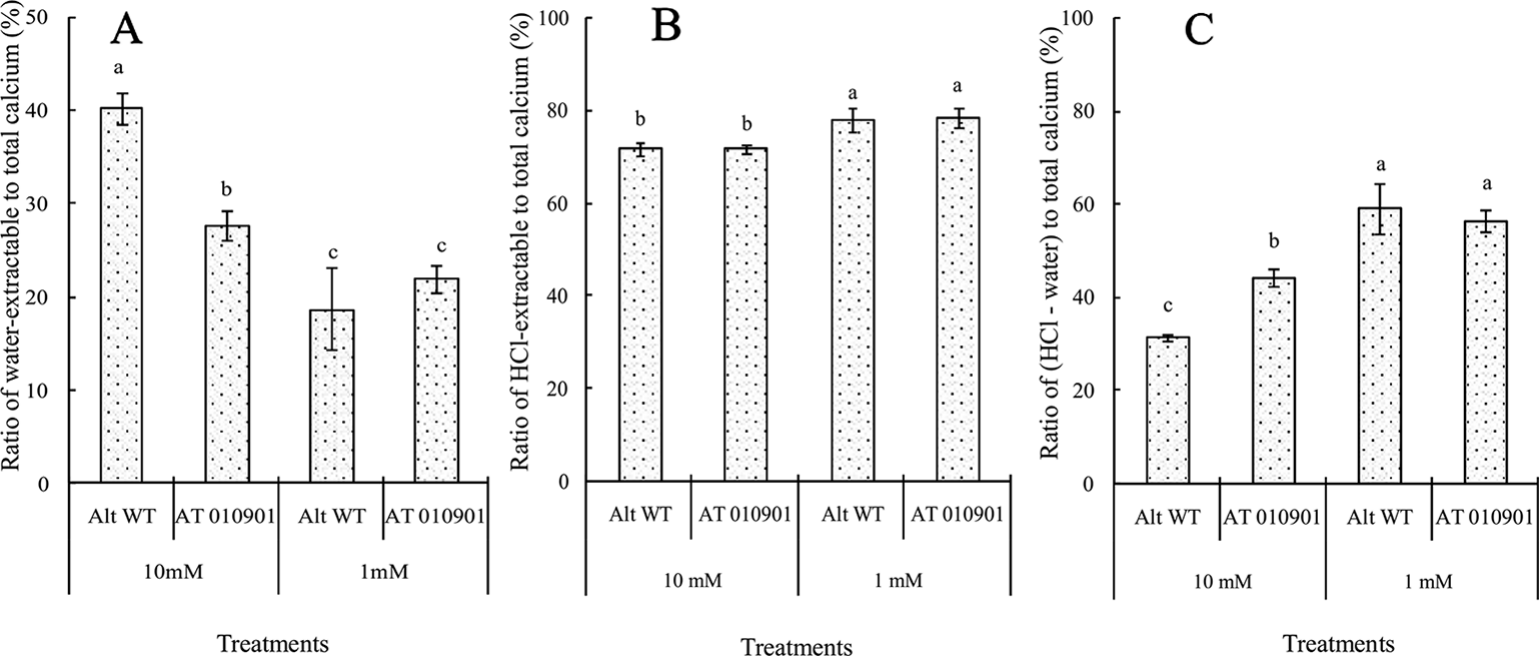
Effects of exogenous calcium on the water-extractable calcium/total calcium **(A)**, HCl-extractable calcium/total calcium **(B)** and (HCl-extractable calcium - water-extractable calcium)/total calcium **(C)** ratios of the mature leaves (fifth fully expanded leaves) of wild-type “Atlantic” plants (WT Atl) and “AT010901” transgenic plants under high-calcium (10 mM) and low-calcium (1 mM) treatments for 3 weeks. The individual data points represent the means ± SEs (n = 5) of five different plants.

The HCl extractable calcium was nearly 78% of the total calcium in both transgenic and wild type leaf tissue at 1 mM calcium treatment (**Figure 6B**) and this decreased slightly to about 75% at 10 mM treatment. On the other hand the water soluble fraction was about 20% of the total calcium fraction at 1 mM treatment and increased to about 26% and 40% at 10 mM calcium treatment in transgenic and wild type plants respectively (**Figure 6A**).

### Changes in Apoplast Calcium Contents in the Mature Leaves of Wild-Type “Atlantic” and Transgenic Plants

**Figure 7:**
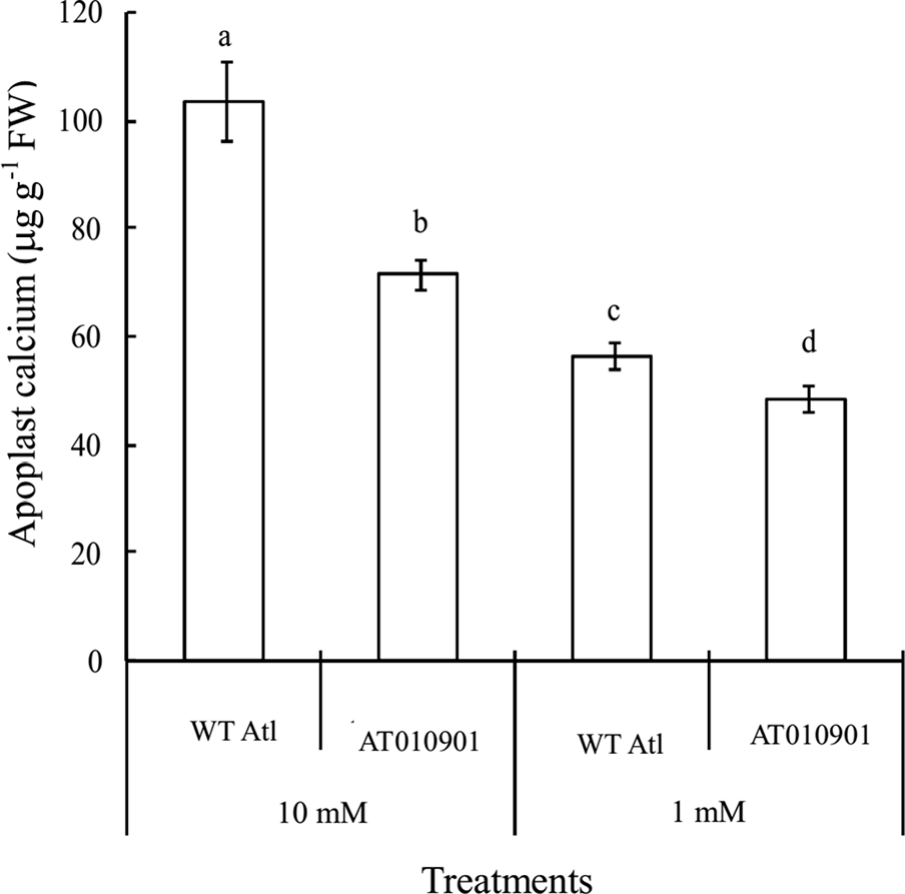
Effects of exogenous calcium on apoplast calcium contents in the mature leaves (fifth fully expanded leaves) of wild-type “Atlantic” plants (WT Atl) and “AT010901” transgenic plants under high-calcium (10 mM) and low-calcium (1 mM) treatments for 3 weeks. The individual data points represent the means ± SEs (n = 5) of five different plants.

The apoplast calcium concentration in the leaf tissue was significantly higher in the wild type as compared to transgenic plants at both 1 and 10 mM calcium treatments (**Figure 7**). Compared with the 1 mM, the 10 mM calcium treatment increased the apoplast calcium content in both wild-type and transgenic plants. Furthermore, the applast calcium concentration in the transgenic plant at 10 mM calcium treatment was greater than the wild type at 1 mM treatment.

### Changes in Calcium Contents in the Tuber Tissue of Wild-Type “Atlantic” and Transgenic Plants

**Figure 8:**
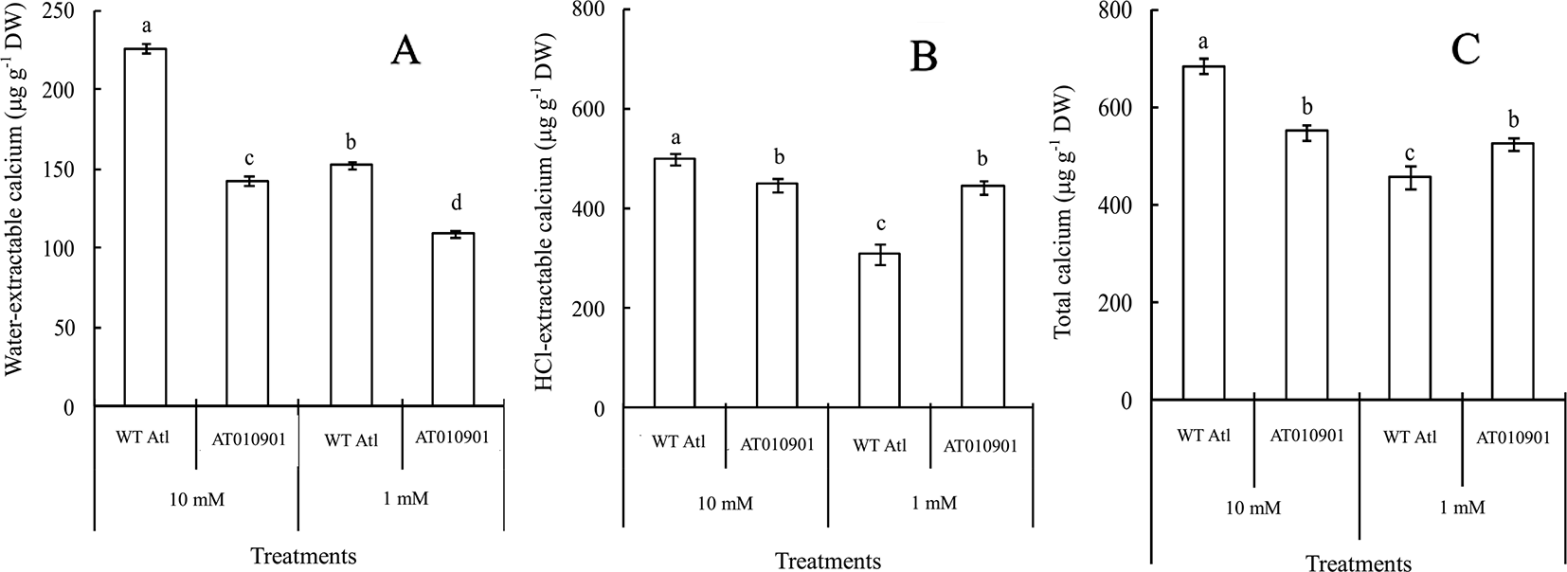
Effects of exogenous calcium on the contents of water-extractable calcium **(A)**, HCl-extractable calcium **(B)** and total calcium **(C)** in the tubers of wild-type “Atlantic” (WT Atl) and “AT010901” transgenic plants under high-calcium (10 mM) and low-calcium (1 mM) treatments for 3 weeks. The individual data points represent the means ± SEs (n = 5) of five different plants.

Tubers of wild type did not show any calcium deficiency symptoms (hollow heart) at either of the calcium nutrition treatments. However, tubers of transgenic line showed hollow heart defects only at 1mM calcium treatment. The water-extractable calcium contents in the tubers of the wild-type ‘Atlantic' plants were significantly greater than those in the transgenic plants at both calcium nutrition treatments (**Figure 8A**). Even though the HCl extractable calcium was higher in transgenic plants at 1 mM calcium treatment, the water soluble calcium was lower in the transgenic plant tubers at this calcium treatment. Furthermore, the 10 mM calcium treatment resulted in a significant increase in water soluble calcium in transgenic tubers. However, the 10 mM calcium treatment did not result in any increase in HCl soluble calcium as compared with 1 mM treatment in the transgenic tubers (**Figures 8B, C**).

**Figure 9:**
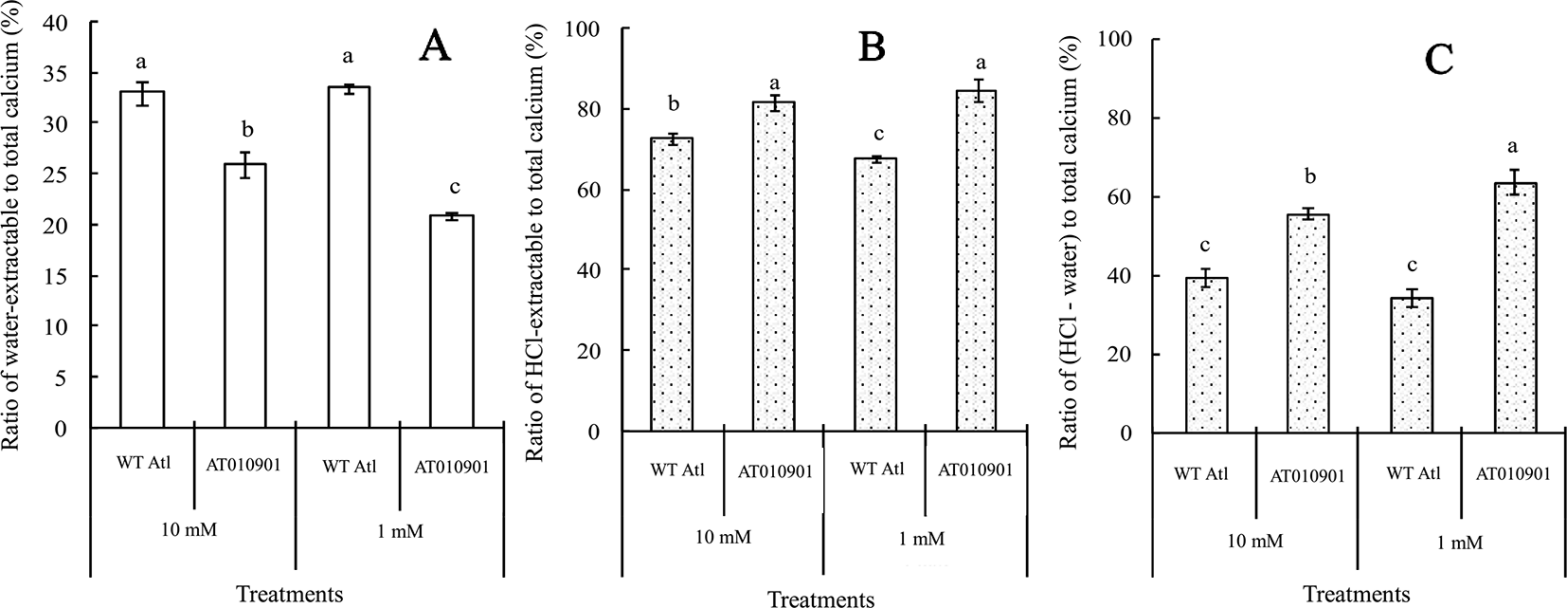
Effects of exogenous calcium on the water-extractable calcium/total calcium **(A)**, HCl-extractable calcium/total calcium **(B)** and (HCl-extractable calcium - water-extractable calcium)/total calcium **(C)** ratios of the tubers of wild-type “Atlantic” plants (WT Atl) and “AT010901” transgenic plants under high-calcium (10 mM) and low-calcium (1 mM) treatments for 3 weeks. The individual data points represent the means ± SEs (n = 5) of five different plants.

Nearly 80% of the total calcium was HCl soluble in the transgenic tubers irrespective of the calcium treatment and this fraction of calcium was significantly lower in the wild type tubers.The water soluble calcium, on the other hand was lower in the transgenic tubers as compared to wild type at both the calcium treatments. Furthermore, the water soluble calcium increased in tubers of transgenic plants at 10 mM calcium treatment as compared to 1 mM calcium treatment. However, in the transgenic plnat tubers there was no change in the HCl soluble calcium in 10 mM calcium treatment as compared to 1 mM calcium treatment. It is interesting to note that the water soluble calcium was much higher proportion of the total calcium in the tuber tissue as compared to leaf tissue (**Figure 9**).

### Changes in Calcium Oxalate Contents in the Cells of Young and Mature Leaves

**Figure 10:**
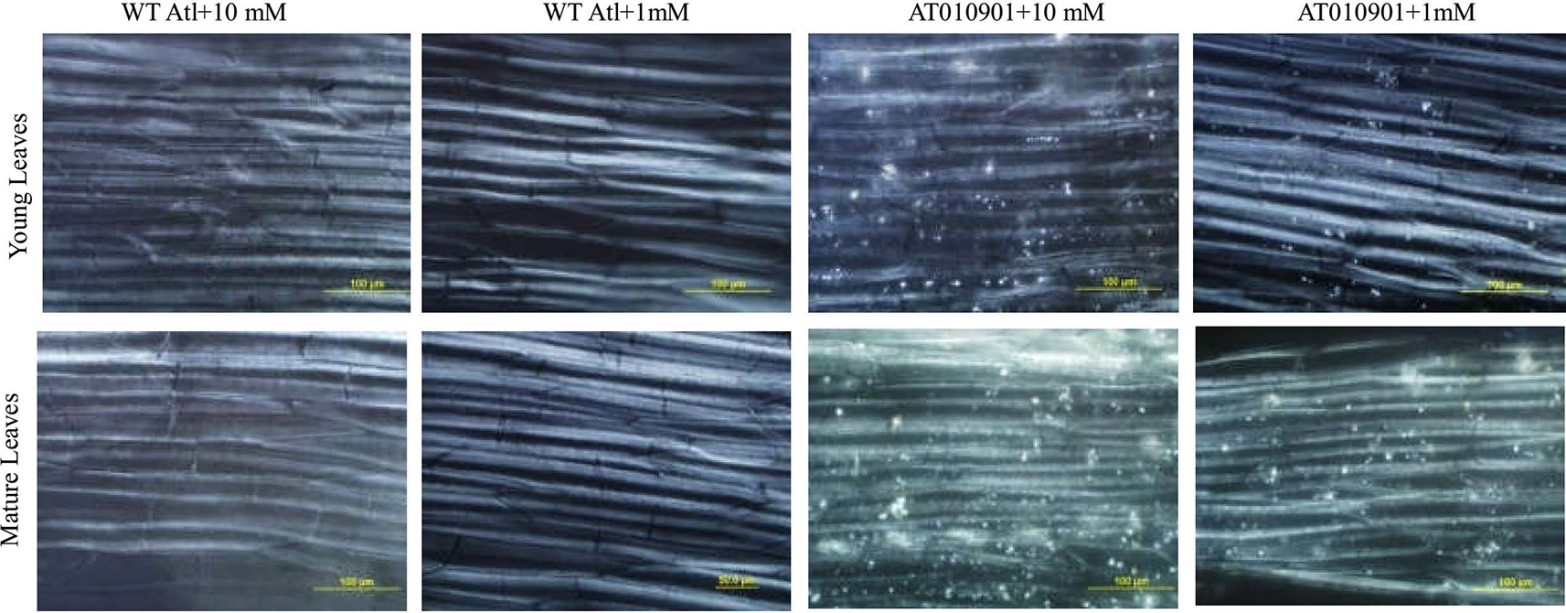
Observations via polarized light of calcium oxalate in the midrib cells of young leaves (second and third expanded leaves) and mature leaves (fifth fully expanded leaves) of wild-type “Atlantic” plants (WT Atl) and “AT010901” transgenic plants under high-calcium (10 mM) and low-calcium (1 mM) treatments for 3 weeks.

Under polarized light, only three-dimensional bi-refringent objects such as crystals refract light in two slightly different directions, which can be detected with a microscope. The results of the polarized light microscopy evaluation of the midrib cells from the young and mature leaves indicate that there are calcium oxalate crystals in the transgenic plants (**Figure 10**). However, these cells in the wild-type plants have only a few or no crystals under both calcium treatments (**Figure 10**). More calcium oxalate crystals in the cells of both the young and mature leaves of the transgenic plants were observed under the 10 mM calcium treatment than under the 1 mM calcium treatment. Moreover, the cells of the mature leaves of the transgenic plants showed more calcium oxalate crystals than did the young leaves under both the 10 mM and 1 mM calcium treatments.

## Discussion

### Overexpression of *sCAX1* Leads to Calcium Deficiency Symptoms in Leaf and Tuber Tissues While Sequestering Calcium in the Vacuole

Results of our study indicate that overexpression of *sCAX1* results in classic symptoms of calcium deficiency in leaves and tubers (**Figure 1**). Transgenic plants given fertilizer solution containing 1 mM calcium daily showed shoot tip damage, leaf curling, leaf necrosis and tuber internal defects (**Figure 1**). These symptoms are known to be related to calcium deficiency in potato (Singh and Sharma, 1972; Kleinhenz et al., 1999; Busse and Palta, 2006; Ozgen et al., 2006; Palta, 2010; Zorrilla et al., 2014).

Nutrient calcium concentration in the range of about 0.1 M is generally considered sufficient for plant health (Hanson, 1984). In our study the transgenic plants showed calcium deficiency when plants were watered daily with nutrient solution containing sufficient calcium (1 mM). Furthermore, the wild type potato plants did not show any of these calcium deficiency symptoms (**Figure 1**). These results are consistent with recent results from our group for transgenic potato lines (Zorrilla et al., 2019). These symptoms occurred even though the total calcium concentration was greater in thetransgenic plants than the wild type in young leaves (**Figure 3**), mature leaves (**Figure 5**) and tubers (**Figure 8**) of the transgenic plants at 1 mM calcium treatment. Thus, the total tissue calcium does not explain the appearance of calcium deficiency symptoms in the transgenic plant. Similar results have been reported for transgenic tomato plants. Tomato transgenic lines overexpressing *sCAX1* had severe symptoms of calcium deficiency known as blossom-end rot even though the total calcium concentration of the fruit was increased as to the wild type (Park et al., 2005a; de Freitas et al., 2011). Furthermore, even though total calcium concentration was increased in the *sCAX1* line grown with 1 mM calcium, the shoot and tuber fresh weight was decreased as compared to the wild type (**Figure 2**). Previous studies also noted necrotic lesions to the plants transformed with *sCAX1*but did not find a reduction in tuber fresh weight (Park et al., 2005b). The differences between our results and those of Park et al. (2005a) could be due to the growing conditions and nutrient management (especially calcium). For example, Park et al. (2005a) conducted their studies in a greenhouse and the calcium applications were made only once a month with 2 mM CaCl_2_; whereas our experiments were conducted in a controlled environment growth chamber and calcium applications were made daily.

In our study, we found calcium oxalate crystals in the leaf cells of transgenic plants at 1 mM calcium treatment (**Figure 10**); however, these crystals were absent in the wild type. More oxalate crystals were detected at 10 mM concentration in the young and mature leaves. These observations are consistent with our recent results on potato transgenic lines overexpressing *sCAX1* (Zorrilla et al., 2019).Over expression of *sCAX1* is expected to increase vacuolar calcium concentration (de Freitas et al., 2011) and vacuolar calcium once stored in the vacuole is generally not redistributed. Excess storage of calcium has been shown to result in the formation of calcium oxalate crystal (Franceschi and Nakata, 2005). Our group has recently shown the presence of these crystals in potato leaf cells overexpressing *sCAX1* (Zorrilla et al., 2019). Results of our study are consistent with those of Zorrilla et al. (2019). These results suggest that even though plants over expressing *sCAX1* have higher total calcium, a portion of this calcium is sequestered in the vacuole in the form of calcium oxalate and thus depleting calcium in the other pools of the cell. This decreased calcium concentration in other pools could result in calcium deficiency symptoms.

### An Increased Supply of Calcium in the Root Zone Relieved Calcium Deficiency Symptoms in the Transgenic Plants

Our results show that calcium deficiency symptoms, that were prevalent in the leaves and tuber tissues at 1 mM calcium treatment, were relieved at 10 mM calcium treatment (**Figure 1**). No internal defects in tubers were detected for the transgenic plants grown in 10 mM Ca nutrient solution. Our previous studies have documented that field grown potato tubers are prone to several calcium deficiency related disorders including internal brown spot, black spot bruising and hollow heart (Palta, 2010). Calcium in plants is known to move with water and in potato plants very little water moves to the tuber because tubers have low transpiration as compared to the leaves (Kratzke and Palta, 1986; Busse and Palta, 2006). Furthermore, an in-season application of calcium around the tuber during the tuber development period has been found to reduce these tuber internal defects (Tzeng et al., 1986; Olsen et al., 1996; Palta, 1996; Kleinhenz et al., 1999; Karlsson et al., 2006; Ozgen et al., 2006). For these defects to be reduced the calcium application to tuber area during the tuber growth is necessary (Palta, 2010). Results of our study suggest the plants overexpressing *sCAX1* need much higher root zone calcium for normal leaf and tuber growth. As discussed above the *sCAX1* overexpressing plants appear to sequester calcium in the vacuole as calcium oxalate and thus making calcium less available to other pools. Thus, as the calcium concentration in the root zone was increased from 1 mM to 10 mM not only the total calcium but also water soluble and HCl soluble concentration was increased. This increased calcium concentration in other pools may have alleviated the calcium deficiency symptoms.

### An Increase in the Water Soluble Calcium and Apoplastic Calcium Appears to Be Related to the Alleviation of Calcium Deficiency Symptoms in the Leaves and Tubers of Transgenic Plants

As indicated above, the calcium deficiency symptoms in the transgenic plants were alleviated when the root zone calcium was increased from 1 to 10 mM. Results of our study indicate that fractionating of calcium in leaf and tuber tissues explain the calcium deficiency symptoms in the *sCAX1* lines at 1 mM calcium. Furthermore, these results also provide insight into the alleviation of the calcium deficiency symptoms in this transgenic line at 10 mM calcium. In addition to total tissue calcium concentration we measured water soluble and HCl soluble calcium in leaf and tuber tissues. We also measured apoplast calcium in leaf tissue. Water soluble fraction is considered to be made up of primarily apoplast calcium (Yoshida et al., 2014). Since calcium oxalate crystal can be dissolved in HCl, the HCl fraction would contain all the water soluble as well as calcium oxalate fractions (Ferguson et al., 1980; Kinzel, 1989). We quantified the water soluble, HCl soluble and total calcium in the leaves and tuber tissues of *sCAX1* grown at 1 mM and 10 mM calcium. Our results show that both in the young and mature leaves all fractions of calcium were increased in the transgenic line as compared to wild type (**Figures 3** and **5**). However, the most dramatic increase was found to be in the water soluble fraction which was 62.5% and 81.7% in the young and mature leaves respectively. These results suggest that lack of adequate water soluble calcium fraction is associated with the calcium deficiency symptoms in the *sCAX1* line grown at 1 mM calcium. Furthermore, an increase in the water soluble calcium is also related to the alleviation of calcium deficiency symptoms in *sCAX1* line grown at 10 mM calcium. In addition, the soluble calcium fraction as the proportion of total calcium, increased from 6% to 9% and from 20% to 40% in the young and mature leaves respectively, in the transgenic line, when calcium in the root zone was increased from 1 mM to 10 mM (**Figures 4** and **6**). These results further show that an increase in water soluble calcium is important in the alleviation of calcium deficiency. In agreement with our results water soluble fraction of calcium has been found to be most significant fraction of calcium related to calcium deficiency in tomato and apple fruits (Pavicic et al., 2004; Yoshida et al., 2014). In agreement with the results on leaf tissue of plants expressing *sCAX1*, the water-soluble calcium was the only fraction that increased, as calcium applied to the root zone increased from 1mM to 10mM (**Figure 8**). These results further strengthen the argument that the water-soluble fraction of calcium plays the most significant role in the health of the tissue.

Analysis of the apoplast calcium fraction also revealed interesting results. There was an increase in the apoplast calcium in the mature leaves when calcium deficiency symptoms were relieved in these leaves at 10mM calcium treatment (**Figure 7**). The *sCAX1*expressing leaves had lower apoplast calcium than the wild type. Furthermore, in this transgenic line apoplast calcium at 10mM treatment was higher than in the wild type at 1mM treatment (**Figure 7**). This means that when the apoplast calcium is above a threshold, the calcium deficiency symptoms are alleviated. In support of this notion early studies indicated that apoplastic calcium concentration must be greater than 0.1 mM to maintain the integrity of the plasma membrane (Clarkson and Hanson, 1980: Hanson, 1984).Our results are also consistent with the results reported by de Freitas et al. (2011) on tomato lines overexpressing *sCAX1*. The fruits on these lines reached 100% blossom-end rot incidence at 15 days after pollination, and the fruit pericarp was found to have lower apoplastic calcium concentration.

Ours is the first study to document the significance of water-soluble calcium in the development of calcium-deficiency symptoms in the potato transgenic lines overexpressing *sCAX1*. Furthermore, our result demonstrates that an increase in this fraction plays a significant role in the alleviation of calcium deficiency symptoms when calcium concentration in the nutrient media is increased. These results provide important insight on the role of *sCAX1* in the calcium homeostasis.

## Data Availability Statement

All datasets generated for this study are included in the article/supplementary material.

## Author Contributions

HG carried out the main work in the manuscript, including the experimental design, plant cultivation, and statistical analysis. JP proposed the research project and statistical analysis method and help write the manuscript until its submission. XW participated in the statistical analyses and revised the manuscript. CZ prepared the experimental materials and participated in the calcium oxalate observations. SV participated in the calcium analysis.

## Conflict of Interest

The authors declare that the research was conducted in the absence of any commercial or financial relationships that could be construed as a potential conflict of interest.

## Abbreviations

cdc2a promoter, cell division cycle promoter; *sCAX1*, cation exchanger 1.
